# Stem Cell Tracking by Nanotechnologies

**DOI:** 10.3390/ijms11031070

**Published:** 2010-03-12

**Authors:** Chiara Villa, Silvia Erratico, Paola Razini, Fabrizio Fiori, Franco Rustichelli, Yvan Torrente, Marzia Belicchi

**Affiliations:** 1 Stem Cell Laboratory, Department of Neurological Sciences, Fondazione IRCCS Ospedale Maggiore Policlinico, Centro Dino Ferrari, Università di Milano, *via* F.Sforza 35, 20122 Milano, Italy; 2 Department SAIFET, Physical Sciences Section, Polytechnic University of Marche, *via* Brecce Bianche, 60131, Ancona, Italy

**Keywords:** stem cells, nanotechnologies, SPIO nanoparticles, X-ray microCT, *in vivo* imaging

## Abstract

Advances in stem cell research have provided important understanding of the cell biology and offered great promise for developing new strategies for tissue regeneration. The beneficial effects of stem cell therapy depend also by the development of new approachs for the track of stem cells in living subjects over time after transplantation. Recent developments in the use of nanotechnologies have contributed to advance of the high-resolution *in vivo* imaging methods, including positron emission tomography (PET), single-photon emission tomography (SPECT), magnetic resonance (MR) imaging, and X-Ray computed microtomography (microCT). This review examines the use of nanotechnologies for stem cell tracking.

## 1. Introduction

**Figure 1.**
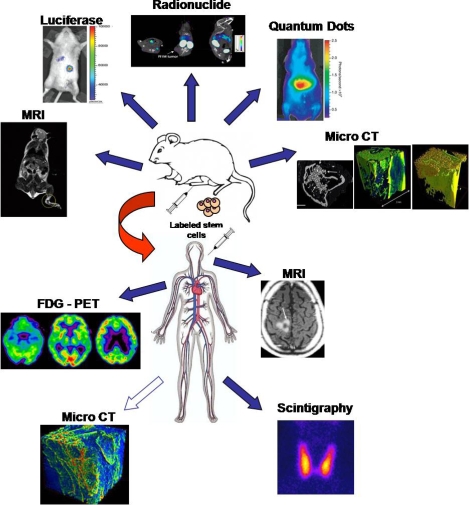
Recent advances in nanotechnology for stem cell tracking. Anatomical and *in vivo* molecular imaging used to assist researchers in locating labeled stem cell. Methods for tracking stem cells in murine animal model such as MRI [56], MicroCT [17], Luciferase [57], Quantum Dot and Radionuclide [58] are shown in the upper panel. MRI and Radionuclide methods are also used in human studies. Improvement and combination of these methods will allow the quantification of migrating stem cells after their systemic use in clinical trials. In particular the future use of Micro-CT *in vivo* in humans should complete the need for new tracking methods (white arrow). Website sources for scintigraphy and FDG-PET: www.ifc.cnr.it; www.pmed.com.

Cells have several advantages as a therapeutic or delivery system: they are able to carry out complex functions and they are responsive to changes in the surrounding tissue of host organism [1–5]. The ability to non invasively monitor cell trafficking *in vivo* in a longitudinal fashion is a pressing need for emerging cellular therapeutic strategies. Monitoring of therapeutic cells is often conducted by histological analyses, which require sacrifice of the animal or tissue biopsies. Recently, non invasive imaging based monitoring methods (Figure 1) have been developed to track stem cell transplantation by labeling injected cells using nanotechnologies [6–15].

The goal is to track the distribution and migration of stem cells once introduced in the model organism. Examples include i) magnetic nanoparticles for stem cell labeling and successive visualization by *in vivo* MRI (Magnetic Resonance Imaging); ii) quantum dots or radionuclides for *in vivo* visualization of stem cells by PET or SPECT. Moreover, the microCT offers high spatial resolution of the distribution of nanoparticles labeled stem cells and provides rapid reconstruction of 3D images and quantitative volumetric analysis. In fact, the fate of injected stem cells in damaged tissues could be monitored by the X-ray micro CT after their labeling with SPIO (SuperParamagnetic Iron Oxide) nanoparticles. The aim of this review is to present some of recent progress obtained by using innovative and non-invasive imaging techniques and nanodiffraction involving nanotechnologies in research areas related to stem cells. In particular, we will focus on the fate of transplanted stem cell labeled with SPIO nanoparticles, as a treatment of muscular dystrophy of Duchenne in small animal models muscle, and tracked *in vivo* using X-Ray microCT. We recently identified a subpopulation of human circulating stem cells which participate actively to muscular regeneration when transplanted in dystrophic animal model migrating through the vasculature [16]. These cells can be labeled with nanoparticles and tracked by microCT [17]. MicroCT imaging is applicable to monitor the stem cell homing, after cell labeling with iron oxide nanoparticles. This technique also offers the possibility of obtaining a quantification of the number of cells that are able to migrate from the blood stream inside the muscle tissue, and a 3D visualization of their distribution and to detect small animal models *in vivo* at several times after the injection.

## 2. Nanoparticles for *in vivo* MRI Visualization of Transplanted Stem Cells

MRI has found extensive applications in stem cell imaging both in research and clinical settings [18–20]. MRI tracking of stem cells has largely relied upon *ex vivo* pre-labeling of stem cells with magnetic nanoparticles which can be internalized by the cells to generate strong MRI contrast [21]. MRI analysis presents a high spatial resolution and the advantage of visualizing transplanted cells within their anatomical surroundings, which is crucial for the description of migration processes. However, the level of sensitivity achieved by this technique is influenced by dilution of contrast agents, due to cell division, or the disposition of some of them to be transferred to non stem cells; in these cases the detected signal decreases and it’s not possible to correlate it to the injected cell number. The recent ability to directly label stem cells with magnetic resonance (MR) contrast agents provides a simple, straight-forward manner to monitor accurately cell delivery and track stem cells non-invasively in a serial manner. A variety of nanoparticles can be constructed to obtain MRI contrast [12,22] and peptide-conjugation approaches can be realized to label cells with multiple-detecting nanoparticles (magnetic, fluorescent, isotope) [23,24]; those currently in use typically range from 5 to 350 nm in diameter. These include superparamagnetic iron oxides (SPIO; 50–500 nm) and ultrasmall superparamagnetic iron oxides (USPIOs; 5–50 nm), which generally are coated with dextran or other polymers to maintain solubility and reduce particle agglomeration. SPIO nanoparticles represents the most widely used contrast agents for the detection of implanted cells *in vivo* because their contrast effect [25,26]. SPIO-labeled stem cells/progenitor cells might contribute to our understanding of cell migration processes in the context of numerous diseases, such as neurologic [27] and muscular diseases [28], myocardial infarction [29–31], and cancer [32]. For example, magnetically labeled mouse embryonic stem cells (mESCs), injected into the nonischemic side of the brain of a rat with partial brain ischemia, can be tracked during their migration along the corpus callosum, populating the border zone of the ischemic area of the contralateral hemisphere [26]. Moreover, SPIO- labeled human neural stem cells can be visualized in mature rodent brain after their transplantationn [27]. In this case, the MRI demonstrated the migration capacity of labeled stem cells into the cortical region of the brain [27]. In addition to the information obtained from cell migration studies, SPIO technology might yield important information about the differentiation process of stem cells/progenitor cells. SPIO-labeled CD34+ progenitor cells injected into rodents can be isolated by magnetic separation after *in vivo* migration to study the differentiation of these cells exposed to a biological environment [33]. A clinical study using stem cells labeled with SPIO in patients with neurological disease has recently been reported [34]. This approach can be adapted to evaluate the therapeutic effects of stem cells in the context of other diseases, including myocardial infarction. In the literature is also reported the use of MRI to monitor the migration of magnetically labeled cells in early phase clinical trials [35]. Autologous dendritic cells were labeled with a clinical superparamagnetic iron oxide formulation or Indium Oxine™ (^111^In oxyquinoline) and were co-injected intranodally in melanoma patient under ultrasound guidance. In contrast to scintigraphy imaging, by using MRI it was possible to serially monitor the migration of these through adjacent lymph nodes. It is important to note that the use of MRI contrast agents for magnet labeling of cells is considered an off-label use of the agent and at this time few superparamagnetic or paramagnetic agents have been approved by regulatory agencies for use to label cells.

## 3. Radioisotope Labeling for PET and SPECT *in vivo* Imaging

The PET and SPECT radionuclide imagine techniques allow the imaging of radiolabeled markers and their interaction with biochemical processes in living animals. Due to their nanomolar (<10^−9^ M) sensitivity, PET and SPECT are able to measure biological process at very low concentrations. The mass of radiotracer injected is extremely small and does not impact the biological system under study. Technological developments of both PET and SPECT have led to the implementation of specialized systems for small animals imaging, with a better spatial resolution (<2 mm) and consequent advancement in the field of cell tracking in animals model *in vivo*.

In general a radioisotope with a relatively long decay half life is use to enable the tracking of cells over period of several hours, or even days (^111^In T ½= 2.8days). Numerous cell tracking experiments have been performed using cells labeled with a radioactive markers. ^111^In-labeled cells have been widely used in humans in localizing areas of inflammation by imaging the leukocyte distribution [36]. Furthermore, ^111^In-labeled cells have been applied in various experimental settings in animals to determine migration of dendritic cells [37], biodistribution of transplanted hepatocytes [38] and even homing of injected mesenchymal stem cells (MSCs) in animal models [39]. Micro-single-photon-emission-computed-tomography (microSPECT/CT) small animal imaging system and an FDA-approved radiotracer (^111^In oxyquinoline), has been use to demonstrate that monocyte recruitment to atherosclerotic lesions. With a noninvasive, dynamic, and three-dimensional fashion in live animals it is possible to track the monocytes recruitment to the atherosclerotic lesions in apolipoprotein E-deficient (ApoE–/–) mice. The long half-life of ^111^In (2.8 days) enabled the detection of monocytes for up to seven days after adoptive transfer, and the high-resolution anatomical data derived from CT allowed localization of hotspots of monocyte infiltration in a sub-millimeter range [40]. Non invasive radionuclide imaging is also well suited to dynamically track the biodistribution and trafficking of mesenchymal stem cells to both target and non target organs. MSCs isolated from bone marrow have the ability to differentiate into multiple cell lineages including osteocytes, chondrocytes, and cardiac myocytes. The recent ability to label MSCs with radiotracers provides a method to serially assess the biodistribution of these stem cells after intravenous administration with the use of radio-nuclide imaging as well as to determine the homing potential of MSCs to sites of injury [30]. The use of the high sensitivity of a combined single-photon emission CT (SPECT)/CT scanner, the *in vivo* trafficking of allogeneic mesenchymal stem cells (MSCs) labeled with a radiotracer and MR contrast agent to acute myocardial infarction was dynamically determined and redistribution of the labeled MSCs after intravenous injection from initial localization in the lungs to nontarget organs such as the liver, kidney, and spleen was observed within 24 to 48 hours after injection. Focal and diffuse uptake of MSCs in the infarcted myocardium was already visible in SPECT/CT images in the first 24 hours after injection and persisted until seven days after injection and was validated by tissue counts of radioactivity. Nevertheless, SPECT- and PET-based tracking of stem cells include nonspecific uptake of the radiotracer by normal tissue, relatively low efficiency of collimated SPECT cameras, and photon attenuation by tissue. Therefore, as concerns ^111^In oxyquinoline ine labeling of human stem cells, the effect of radiation dose on human cell lines need to be carefully observed. A critical factor is to determine whether the ^111^In oxyquinoline labeling affects viability, functionality, migration and proliferative capacity in distinct cell populations as well as species.

## 4. Quantum Dots for Labeling Of Stem Cells

Recent advances in nanotechnology offer some prospects to combine the best of each imaging technique with respect to sensitivity and specificity. There is now an array of artificial particulate systems used as diagnostic agents capable of targeting different cells *in vivo*. Those include colloidal gold, superparamagnetic iron-oxide crystals, dendrimers, polymeric micelles and liposomes, nanotubes, nanowires, nanoshells, and quantum dots (QDs). Quantum dots consist of semiconductor nanocrystals 2–5 nm in diameter, which have highly favorable fluorescence properties (broad band absorption spectra, narrow band emission, and high resistance to photobleaching) compared to commonly used fluorophores [41]. Fluorescent QDs possess several unique optical properties best suited for *in vivo* imaging [42, 43]. Because of quantum confinement effects, the emission color of QDs can be precisely tuned by size from the ultraviolet to the near-infrared. QDs are extremely bright and photostable. Colhera toxin subunit B (CTB)-quantum dots conjugates were developed for labeling mammalian cells. Several stem cell types were labeled with CTB-QD conjugates and quantum dots were completely dispersed throughout the cytoplasm in each cell type, presumably in vesicles [44]. Stem cells labeled appear to maintain their differentiation potential as well as stem cell properties [44]. CTB-QD labeled muscle derived stem cells maintain similar percentage of expression for surface markers indicative of stem cell phenotype, such as stem cell antigen 1 (sca-1) and CD34. They can also form myotubes under serum deprivation, hence maintaining their myogenic potential following labeling with CTB-QD conjugates. The CTB-QD conjugates are likely to be suitable for long term cell tracking [44]. Functionalized quantum dots offer several advantages for tracking the motion of individual molecules on the cell surface, including selective binding, precise optical identification of cell surface molecules, and details examination of the molecular motion. They were conjugated with integrin antibodies to perform studies about changes in the integrin dynamics during osteogenic differentiation of human bone marrow derived progenitors cells (BMPCs) [45]. It was possible to obtain a single particle tracking, by which it was possible to monitor and determine quantum dots conjugated integrin molecules on the surface of BMPC and to elucidate the physical constraints on the protein mobility at the cell surface.

QDs long-term fluorescence makes them an important new class of nanomaterials available for stem cell tracking advance. Although QDs can be optically imaged, *in vivo* tracking typically requires a whole animal imaging. None of whole animal imaging systems have been employed so far to track QDs labeled stem cells *in vivo*. Moreover, QDs size and surface coating might affect their cellular internalization, their intracellular concentration and, consequently, the cytotoxicity of QDs.

## 5. Nanotechnologies and MicroCT as a New Method for *in vivo* Cell Tracking

MicroCT is similar to conventional CT systems usually employed in medical diagnoses and industrial applied research, but unlike these systems, which typically have a maximum spatial resolution of about 0.5 mm, advanced microCT is capable of achieving a spatial resolution up to 0.3 μm, about three orders of magnitude lower. Use of synchrotron X-rays has several advantages compared to laboratory or industrial X- ray sources, such as high spatial resolution and a wide range of greyscale values (corresponding to different X-ray absorption coefficients) within and among datasets [46].

More recently Synchrotron Radiation (SR) microCT systems were made available also for imaging small animals *in vivo*, such as for the examination of living rats [47] or mice [48]. The great advantage of such systems is to enable longitudinal studies, thus reducing the effect of biological variability in the cohort. The first *in vivo* longitudinal study reported alterations of bone micro-architecture in the hind limb loaded female rats [49]. *In vivo* microCT was also used to monitor microarchitectural changes in ovariectomized rats at the tibial metaphyses [50, 51]. It is a non-invasive technique giving integral information about the content of magnetic material along the beam direction as well as a relative local snapshot of the magnetic nanoparticle distribution in relation to the number of slices [52]. MicroCT provides high spatial resolution images (from 10 μm to 1 μm) with high signal-to-noise ratio. In previous study [17] we showed that X-ray microCT analysis is able to detect stem cells, previously labeled with nanoparticles of iron oxide (Endorem^®^), inside skeletal muscles of dystrophic mice after intra-arterial transplantation, providing biological insights into the early processes of muscle stem cell homing.

We explored the use of X-ray microCT as an experimental technique with high spatial resolution of 1.65 μm for detection of stem cells. This technique also offers the possibility of obtaining a quantification of the number of cells that are able to migrate from the blood stream inside the muscle tissue, and a 3D visualization of their distribution. We analyzed nine muscular biopsies transplanted with three different numbers of stem cells labeled with iron-oxide nanoparticles, at three different times after injection. The different timing investigated did not show differences in the location of stem cells, while the variation in stem cells number allowed us to optimize the experimental conditions and identify 50,000 as the minimum number of detectable cells in a murine muscle. We showed that X-ray microCT offers the possibility to detect with high definition and resolution human cells after transplantation, and opens new possibilities for stem cell research. In the perspective of clinical translation of stem cell research, it would be advantageous to develop new techniques to detect donor cells after transplantation to track their fate *in vivo*.

## 6. Conclusions

For clinical efficacy, it is imperative to image stem cells and their final location *in vivo*. Detection by MRI of magnetic nanoparticle-labeled stem cells may serve as a suitable means to achieve this objective; however, this technique has some limitations, such as the gradual loss of MRI cell signal due to cell division. It is also difficult to correlate the magnetic resonance signals to the number of cells detected. QDs might be an alternative for the long-term labeling of stem cells. However, the current cost of QDs labeling and accessibility of whole animal imaging is a barrier to large-scale studies.

Further, quantum dots are not completely innocuous but they can induce alterations in the differentiation profile of stem cells [53,54], and abnormalities during embryonic development [55]. The luciferase based bioluminescence imaging technique has been extensively applied for non invasive imaging and for studying *in vivo* cells trafficking. However, several problems arose concerning the numbers of cells needed to develop *in vivo* experiments and also the quality of the images visualized. With the current advances in stem cells research, microCT imaging, as a non invasive technique, could be applied for tracking the transplanted stem cells and may be an important tool for monitoring the efficacy of stem cells transplantation.

Combining the nanoparticles cell labeling and the X-ray microCT, it is possible to provide detailed information on the stem cell migration in 3D, which is not attainable by traditional methods based on 2D techniques such as histology, scanning electron and fluorescence microscopy imaging. The microCT imaging could be applied on investigations of the homing specificity of various stem cell subtypes or genetically engineered stem cells in different cell-based therapies. We are going to explore the use of X-ray microCT with high spatial resolution of 7.5 μm for detection of stem cells *in vivo*. In particular, we labeled human CD133+ cells with nanoparticles of iron oxide and we injected them intra-arterially into skeletal muscles of dystrophic mice. We already were able to successfully detect stem cells in living mice at different time points. We know that these stem cells can migrate and they have the maximum migration rate in two hours after intra-arterial engraftment into scid/mdx mice, reaching a “plateau” after that time. Further studies are in progress to quantify the number of the stem cells migrated and more analyses are necessary to improve this method for future applications in the human tissues.
